# Abscisic Acid and Abiotic Stress Tolerance in Crop Plants

**DOI:** 10.3389/fpls.2016.00571

**Published:** 2016-05-04

**Authors:** Saroj K. Sah, Kambham R. Reddy, Jiaxu Li

**Affiliations:** ^1^Department of Biochemistry, Molecular Biology, Entomology and Plant Pathology, Mississippi State UniversityMississippi State, Mississippi, MS, USA; ^2^Department of Plant and Soil Sciences, Mississippi State UniversityMississippi State, Mississippi, MS, USA

**Keywords:** abiotic stress, abscisic acid, ABA signaling, gene expression, phytohormone

## Abstract

Abiotic stress is a primary threat to fulfill the demand of agricultural production to feed the world in coming decades. Plants reduce growth and development process during stress conditions, which ultimately affect the yield. In stress conditions, plants develop various stress mechanism to face the magnitude of stress challenges, although that is not enough to protect them. Therefore, many strategies have been used to produce abiotic stress tolerance crop plants, among them, abscisic acid (ABA) phytohormone engineering could be one of the methods of choice. ABA is an isoprenoid phytohormone, which regulates various physiological processes ranging from stomatal opening to protein storage and provides adaptation to many stresses like drought, salt, and cold stresses. ABA is also called an important messenger that acts as the signaling mediator for regulating the adaptive response of plants to different environmental stress conditions. In this review, we will discuss the role of ABA in response to abiotic stress at the molecular level and ABA signaling. The review also deals with the effect of ABA in respect to gene expression.

## Introduction

The world population is increasing and is projected to rise by more than one billion by 2030 and over 2.4 billion by 2050 (United Nations Department of Economic and Social Affairs, and Population Division, 2015). Therefore, to feed the increasing population, agricultural food production must be increased by 70 percent by 2050 (Tilman et al., 2011; Wani and Sah, 2014). The current and projected changes in abiotic stresses such as drought, salinity, cold, and heat will adversely affect the plant growth that ultimately limits the productivity and is the leading cause of crop losses worldwide (Boyer, 1982; Tuteja, 2007; Qin et al., 2011; Bailey-Serres et al., 2012), and thus our ability to feed the population.

According to various studies, abiotic stresses trigger many physiological, biochemical, and molecular responses that influence various cellular processes in plants (Wang et al., 2001, 2003; Hasanuzzaman et al., 2013). To combat various environmental stresses novel and dynamic approaches should be devised, and phytohormone engineering could be a method of choice to improve the productivity. Phytohormones are the key regulators of plant growth and development as well as mediators of environmental stress responses (Sreenivasulu et al., 2012). Among various phytohormones, abscisic acid (ABA), which is the central regulator of abiotic stress resistance in plants and coordinates an array of functions (Finkelstein, 2013; Wani and Kumar, 2015), enabling plants to cope with different stresses. In the plant, when environmental conditions are harsh, the level of ABA increases via ABA biosynthesis. The increased ABA binds to its receptor to initiate signal transduction leading to cellular responses to stresses (Ng et al., 2014); therefore, ABA is also called a stress hormone (Mehrotra et al., 2014). ABA is a weak acid that was first isolated from young cotton fruits as an abscission-accelerating substance by Frederick Addicott and his associates (Ohkuma et al., 1963). Initially, it was thought that ABA had a significant role in the process of fruit abscission (Ohkuma et al., 1963; Cracker and Abeles, 1969). However, later intensive studies show that ABA was not directly involved in abscission process. The precise role of ABA in abscising organs is to promote senescence and stress responses, which are the processes preceding abscission (Finkelstein, 2013). The environmental stresses like drought, salt, and low temperature that lead to decreased water availability (Cutler et al., 2010; Kim et al., 2010), also called osmotic stress, promote the synthesis of ABA. ABA is significantly increased under drought or salinity stress conditions, stimulating stomatal closure, change in gene expression, and adaptive physiological responses (Seki et al., 2002; Yamaguchi-Shinozaki and Shinozaki, 2006; Shinozaki and Yamaguchi-Shinozaki, 2007; Cutler et al., 2010; Kim et al., 2010). ABA also plays an important role in many cellular processes including seed development, dormancy, germination, vegetative growth (Xiong and Zhu, 2003; Finkelstein et al., 2008) and modulation of root architecture (Harris, 2015). Since the discovery of ABA, several efforts have been devoted to understanding how ABA is synthesized under stress conditions.

For ABA perception and signaling, two breakthroughs were achieved in 2009 by the discoveries of the soluble ABA receptor proteins and the core signaling complexes that perceive ABA and transmit cues to subsequent molecular events (Fujii et al., 2009; Ma et al., 2009; Park et al., 2009), which adds the essences of more research with new ideas in ABA signaling. Recently, Park et al. (2015) engineered ABA receptor using agrochemicals, which provide new possibilities for the better development of the crop. Thus, a better understanding of ABA regulatory mechanisms will contribute to engineered stress tolerant crop plants, which is one of the primary goals of plant molecular biologists. This review focuses on the recent development of the role of ABA in understanding cellular networks of biotechnological relevance in abiotic stress responses of crop plants.

## Role of ABA in Plants

Abscisic acid is the most important phytohormone that confers abiotic stress tolerance in crop plants (Shinozaki and Yamaguchi-Shinozaki, 2000; Schroeder et al., 2001). In stress conditions like drought, extreme temperature, and high salinity, content in plants increases considerably, inspiring stress-tolerance effects that help plants, adapt, and survive under these stressful situations (Ng et al., 2014). ABA is also required for plant growth and development under non-stress conditions. ABA has multiple functions in plants. Among them, primary features are as follows.

### Seed Dormancy and Germination

Seed is a crucial organ in higher plants and the transition to seed dormancy and germination signifies a key stage in the plant life cycle, which is an important ecological and economical traits. Two hormones play a role in controlling the mechanism of seed dormancy and germination, i.e., ABA and gibberellins (GAs). Both hormones, ABA, and GAs monitor the equilibrium between seed dormancy and germination. The ABA plays a central role in the induction and maintenance of seed dormancy. It also inhibits the transition from embryonic to germination growth. During the phase of desiccation tolerance, ABA metabolism must be regulated. Sometimes, ABA translocates from the roots through xylem or phloem (Rodríguez-Gacio et al., 2009; Miransari and Smith, 2014) to the shoot system to exert its action there.

### Modulation of Root Architecture

In roots, three main factors control the architecture of roots, i.e., lateral root (positions of branch roots), the angle form with parent root, and root length. Root structure is determined through interactions between the roots and its environment during their lifetime (Harris, 2015). One of the primary functions of ABA is altering root architecture, and thus changing the pattern of growth and quiescence in plant roots. For the root system, the major abiotic stress occurs when there is a scarcity of water, or the availability of water is inconsistent. Under this scenario, the levels of ABA is altered in response to the intensity of water stress. Subsequently, changes in the root environment will have both local and systemic effects on ABA-mediated responses (Dodd et al., 2008; Dodd et al., 2010; Puertolas et al., 2015). Similarly, ABA is an important mediator of drought, salt, and osmotic stress. All these three stresses reduce soil water potential whereas, salt stress has the added the component of ionic stress. Both salt and osmotic stresses lead to increased osmotic strength.

ABA stimulates water flow and ion flux in root tissues suggesting that ABA regulates turgor by decreasing transpiration as well as by increasing water influx into roots (Glinka and Reinhold, 1971). In the presence of ABA, at low water potentials, the ratio of root growth to shoot growth is higher than in the absence of ABA (Saab et al., 1990). Similarly, Spollen et al. (2000) also reported that, at low water potential, ABA accumulation maintains maize primary root elongation by restricting the production of ethylene and demonstrated that ethylene plays a main role in inhibition of root elongation. At low water potential, the maize primary root became thinner and adaptive toward resource utilization by searching new soil for water (Sharp et al., 1988; Liang et al., 1997), whereas ethylene increases lateral expansion or roots. It is also speculated that later root-specific activation of ABA signaling will shift the balance of root growth toward soil exploration, away from resource utilization (Duan et al., 2013). The accumulation of ABA in maize roots helps to elongate the roots, allowing for the exploitation of surrounding soil environments and contributing to the plants ability to cope of with water stress.

Also, ABA may act in root meristem maintenance (Liang et al., 2007). The evidence was supported by Zhang et al. (2010a), reported that ABA can promote the maintenance of stem cell by promoting of QC (quiescent center) quiescence and the suppression of stem cell differentiation (Ortega-Martínez et al., 2007; Sarkar et al., 2007; Zhang et al., 2010a). All the effects of ABA on roots appear to modulate root architecture to adopt the environmental stress conditions.

### Senescence

Leaf senescence is an essential part of the final stage of plant development, which is regulated by a complex range of endogenous and environmental factors (van der Graaff et al., 2006; Lim et al., 2007). Among these factors, ABA has been put forward to affect leaf senescence significantly (Xue-Xuan et al., 2010). Foliar spraying with ABA has been revealed to promote leaf senescence in rice (Ray et al., 1983) and similar results were shown in maize by He and Jin (1999). In another study, He et al. (2005) reported that the translocation of ABA from roots to shoots may be blocked in the stay green cultivar (Maize P3845) in which lower ABA level might be significant for delaying leaf senescence. Further, it has been reported that with ABA can induce leaf yellowing, which is an indicator of leaf senescence (Yang et al., 2003; Fang et al., 2008).

An increase in endogenous ABA appears to coincide with senescence of leaves (Yang et al., 2002), which supports the notion that ABA functions in the leaf senescence. Likewise, ABA can promote senescence and induce expression of several specific senescence-associated genes (Xue-Xuan et al., 2010; Finkelstein, 2013). Many studies also reported that, in *Arabidopsis* mutants with deficiencies in ABA biosynthesis or signaling, exhibit altered or delayed senescence (Gan, 2003; Lim and Nam, 2005; Passioura, 2007). Lee et al. (2011) identified a receptor kinase (RPK1) that mediates age-and ABA-induced senescence in old leaves. The four different independent studies reported that NAP (NAC-like, activated by apetala3/pistillata) is a significant positive regulator that controls leaf senescence in rice and *Arabidopsis* (Guo and Gan, 2006; Zhou et al., 2013; Liang et al., 2014; Yang et al., 2014). Moreover, AtNAP, an ABA-inducible NAC family transcription factor, has been shown to play a fundamental role in leaf senescence (Guo and Gan, 2006). Zhang and Gan (2012) reported that senescence-associated gene 113 (*SAG113*) positively regulates ABA-induced leaf senescence by inhibition of stomata closure, leads to accelerates water loss in senescing leaves. In rice, overexpression of an NAC-like gene (*OsNAP*) can promote leaf senescence while knockdown of *OsNAP* delay leaf senescence (Liang et al., 2014). Further, the expression of *OsNAP* can be induced by ABA and is reduced in ABA-deficient mutants. They concluded that ABA-mediated leaf senescence is dependent primarily on the modulation of *OsNAP* expression and that OsNAP controls the ABA synthesis via a feedback mechanism (Liang et al., 2014). Recently, Takasaki et al. (2015) reported that SNAC-A, a subfamily of stress responsive NAC transcription factors, plays critical roles in ABA-induced leaf senescence signaling in *Arabidopsis* (Takasaki et al., 2015). Similarly, a cotton NAP-like transcription factor (GhNAP) has been shown to regulate leaf senescence through ABA-mediated pathways (Fan et al., 2015). All these studies point to the significance of NAC-type transcription factors in ABA-mediated leaf senescence.

### Stomata Regulation

Stomata are small pores on the leaf surfaces formed by guard cells, which control plant gas exchange processes. Light usually stimulates stomatal opening whereas ABA and elevated CO_2_ levels promote partial or complete closure of stomata (Kim et al., 2010). During stomatal closure, decreased gas exchange results in the reduction of photosynthate production while decreased transpiration can reduce water loss from leaves (Suzuki et al., 2013; Mittler and Blumwald, 2015). In drought conditions, ABA alteration of guard cell ion transport, which promotes stomatal closure and prevents stomatal opening, reducing water loss (Kim et al., 2010). The ABA-activated protein kinase (AAPK) from *Vicia faba* is a guard cell-specific protein kinase whose catalytic activity is activated by ABA (Li and Assmann, 1996). Further, Li et al. (2000) showed that AAPK is a positive regulator of ABA-induced stomatal closure through activating plasma membrane anion channels. Many studies have shown that two types of anion channels that mediate anion release from guard cells, activated by elevated cytosolic Ca^2+^ levels, i.e., slow-acting sustained (S-type) and rapid transient (R-type) (Schroeder and Keller, 1992; Schroeder et al., 2001). Only S-type channel is responsible for the ABA-mediated stomatal closure (Joshi-Saha et al., 2011). Two different groups reported that Slow Anion Channel-Associated 1 (SLAC1), is the S-type channel that triggers membrane depolarization required for stomatal closure (Negi et al., 2008; Vahisalu et al., 2008). The OST1 (open stomata 1) kinase is an AAPK-related protein kinase that mediates ABA-induced stomatal closure in *Arabidopsis* (Mustilli et al., 2002). Recently, Grondin et al. (2015) identified OST1 as a novel regulator of plasma membrane intrinsic proteins (PIPs, a type of plant aquaporins) through phosphorylating a distinct phosphorylation site. They performed assays in epidermal peels which showed that *pip2;1* knockout mutants in *Arabidopsis* have a defect in stomatal closure in response to ABA. This study has determined the novel function of aquaporins, which contribute to ABA-triggered stomata closure that requires an increase in guard cell permeability to water via OST1 dependent phosphorylation of PIP2;1 at Ser-121 (Grondin et al., 2015). Several studies revealed that activated OST1 kinase binds directly to and phosphorylates the anion channel SLAC1, intervening anion release from the guard cells and promoting stomatal closure (Geiger et al., 2009, 2010; Lee et al., 2009; Brandt et al., 2012). Similarly, Sato et al. (2009) reported that K^+^ channel KAT1 is also a target of SnRK2.6/OST1 and phosphorylation occurs in the C-terminal region of KAT1. These results suggest that phosphorylation is important for regulating ion channels. Zou et al. (2015) reported that the functions of CALCIUM-DEPENDENT PROTEIN KINASE8 (CPK8) in ABA-mediated stomatal regulation in response to drought stress via regulating CATALASE3 (CAT3) activity. They also reported that CPK8 can phosphorylate CAT3 at Ser-261 and regulate its activity (Zou et al., 2015). Constant eﬄux of both anions and K^+^ from guard cells drives water eﬄux and contributes toward to the loss of guard cell turgor, leading to stomatal closure (Fan et al., 2004; Munemasa et al., 2015). For depolarization of the guard cell membrane, ABA also inhibits the activity of guard cell plasma membrane H^+^-ATPase (Hayashi et al., 2011). ABA can induce the accumulation of reactive oxygen species (ROS) in guard cells, causing stomata to close. Two NADPH oxidases, *AtrbohD*, and *AtrbohF* are responsible for ABA-promoted ROS production in *Arabidopsis* guard cells (Kwak et al., 2003). OST1 can phosphorylate AtrbohF, presumably affecting its enzymatic activity important for ROS production (Sirichandra et al., 2009).

Reactive oxygen species could enhance ABA biosynthesis (Zhao et al., 2001). ABA treatment enhances the ROS production in guard cells (Pei et al., 2000). Some studies also suggested that ROS accumulation is involved in the induction of stomatal closure (An et al., 2008; Khokon et al., 2011). Thus, enhanced ROS production in the guard cells creates a positive feedback loop to mediate stomatal closure (Mittler and Blumwald, 2015). The level of ABA in guard cells can increase by *de novo* synthesis, transporter-mediated import, and recycling from inactive conjugates (Merilo et al., 2015). Rapid stomatal responses to environmental stimuli might mainly rely on guard cell-synthesized ABA whereas ABA synthesized in the vasculature might contribute more to stomatal regulation during long-term soil water deficit (Merilo et al., 2015). In this way, ABA has a very useful role in regulating stomatal aperture, which helps plants to adapt and survive stress conditions.

### Abiotic Stress Response

Abscisic acid is believed to be the key hormone that mediates plant responses to adverse environmental stimuli since the level of ABA in plants usually increases during abiotic stress conditions, and elevated ABA can enhance plant adaptation to various abiotic stresses (Swamy and Smith, 1999; Tuteja, 2007). Since the first observation of ABA accumulation in drought-stressed wheat (Wright, 1969), increased levels of endogenous ABA under drought stress conditions have been reported in many plant species which include maize (Beardsell and Cohen, 1975; Wang et al., 2008), sorghum (Kannangara et al., 1983), rice (Henson, 1984), barley (Stewart and Voetberg, 1985; Thameur et al., 2011), soybean (Bensen et al., 1988), and wheat (Guoth et al., 2009). Some earlier studies have shown endogenous ABA accumulation in response to cold stress (Daie and Campbell, 1981; Eze et al., 1983; Lalk and Dörﬄing, 1985). Increased level of endogenous ABA has also been observed in salt-stressed tobacco cells and alfalfa seedlings (Singh et al., 1987; Luo et al., 1992). Exogenous application of ABA to plants can increase their adaptive responses to abiotic stresses. External application of ABA has been revealed to increase drought tolerance in some plant species (Waterland et al., 2010; Du et al., 2013; Yadegari et al., 2014; Wei et al., 2015). ABA treatments could increase cold resistance in cucumber (Flores et al., 1988) and alfalfa (Mohapatra et al., 1988). It is also shown that ABA can alleviate salt stress in common bean and potato (Khadri et al., 2006; Etehadnia et al., 2008).

In addition to its role in regulating stomatal aperture, which is required to limit water loss from leaves under drought conditions, ABA induces the expression of many genes whose products are important for stress responses and tolerance such as enzymes for osmoprotectant synthesis (Fujita et al., 2011). Transcriptome studies have shown that over 50% of the genes regulated by ABA are also governed by drought or salinity, whereas cold-regulated transcriptome shows less overlap with those induced by other stresses. Seki and his associates identified 245 ABA-inducible genes in *Arabidopsis*. Among the ABA-inducible genes, 63% (155 genes) were induced by drought, 54% (133 genes) by high salinity, 10% (25 genes) by cold treatment (Seki et al., 2002). Rabbani et al. (2003) identified 73 stress-inducible genes in rice, among them, 43 genes were induced by ABA. These results indicate significant crosstalk between ABA response and abiotic stress signaling pathways, especially for drought and high-salinity.

The main goal of studying plant stress responses is to develop crops with improved tolerance to abiotic stresses. The knowledge of ABA being a key regulator of abiotic stress responses has been utilized for developing crops with enhances tolerance under stress conditions. Molecular manipulation of ABA synthesis or signaling has been done in different crop plants for improved stress tolerance (**Table 1**). Transgenic crops overexpressing the key ABA synthesis gene *NCED* (9-*cis*-epoxycarotenoid dioxygenase) have been shown to exhibit improved drought tolerance (**Table 1**). Similarly, crops overexpressing the ABA receptor gene PYR (pyrabactin resistance) have been reported to confer drought tolerance (**Table 1**). However, in many studies, the effects of ABA manipulated transgenics were not tested for its performance in yield though they reported tolerance toward abiotic stress. Some studies reported improved biomass and nutritional quality at yield level (Sivamani et al., 2000; Aswath et al., 2005; Bao et al., 2016). Wang et al. (2009) reported that RNAi-mediated suppression of the alpha subunit of protein farnesyltransferase (protein farnesyltransferase had been shown to be a key negative regulator controlling ABA sensitivity in *Arabidopsis* guard cells; Pei et al., 1998) leads to drought tolerance in canola under drought conditions. In well water conditions, they were not able to find yield difference but in drought conditions, they found better yield than non-transgenics. Recently, Wang et al. (2016) reported that the major latex protein-like protein 43 (MLP43) is a positive regulator of ABA response and overexpression of *MLP43* conferred drought tolerance in *Arabidopsis*. It remains to be seen whether genetic manipulation of *MLP43* expression could improve the drought tolerance of crops.

**Table 1:** List of transgenic plants that used genes for improved abiotic stress tolerance through manipulating ABA signaling or synthesis in crops.

Gene transferred/targeted	Gene product	Transgenic plant	Abiotic stress	Yield^∗^	Reference
*PYR/PYL/RCAR*	ABA receptors	Tomato	Drought	NA	Gonzalez-Guzman et al., 2014
*OsPYL3 and OsPYL9*	ABA receptors	Rice	Drought and cold	NA	Tian et al., 2015
*SIAREB1*	AREB/ABF subfamily of bZIP transcription factors	Tomato	Drought and salt	NA	Orellana et al., 2010
*OsbZIP46CA1*	bZIP transcription factor	Rice	Drought and osmotic stress	NA	Tang et al., 2012
*OsbZIP72*	bZIP transcription factor	Rice	Drought	NA	Lu et al., 2009
*OsSLI1*	Homeodomain-leucine zipper type I	Rice	Multiple abiotic stresses and panicle development	NA	Huang et al., 2014
HVA1	ABA-responsive late embryogenesis abundant protein	Wheat	Drought	Improved biomass	Sivamani et al., 2000
BnFTA	Farnesyl transferase	Canola	Drought	Improved during drought conditions	Wang et al., 2009
*LeNCED1*	9-*cis*-epoxycarotenoid dioxygenase	Tomato	Drought	NA	Thompson et al., 2000
PvNCED1	9-*cis*-Epoxycarotenoid Dioxygenase	Tobacco	Drought	NA	Qin and Zeevaart, 2002
VuNCED1	9-*cis*-epoxycarotenoid dioxygenase	Bent grass	Drought and salt	Biomass increased with 3–4 fold as compared to control	Aswath et al., 2005
*SgNCED1 and ALO*	co-expressing stylo 9-*cis*-epoxycarotenoid dioxygenase and yeast D-arabinono-1,4-lactone oxidase genes	Tobacco	Drought and chilling	Improved nutritional quality, i.e., vitamin C	Bao et al., 2016
*SgNCED1*	9-*cis*-epoxycarotenoid dioxygenase	Tobacco	Drought and salinity	NA	Zhang et al., 2008a, 2009
*LeNCED1*	9-*cis*-epoxycarotenoid dioxygenase (*NCED*)	Petunia	Drought	NA	Estrada-Melo et al., 2015

*^∗^NA, not applicable*.

## ABA Biosynthesis, Catabolism, and Transport

### ABA Biosynthesis

**FIGURE 1:**
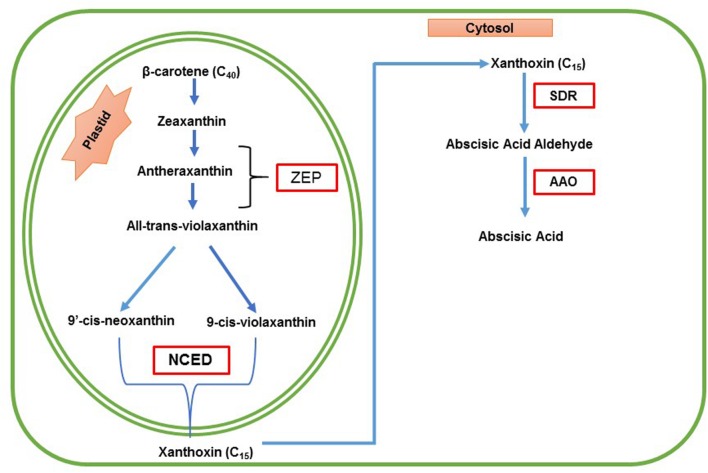
**Schematic representation of biosynthesis of ABA in plants.** ABA is derived from β-carotene (C_40_) through an oxidative cleavage reaction in plastids. The first step of ABA biosynthesis pathway is the conversion of zeaxanthin and antheraxanthin to all trans-violaxanthin, which will be catalyzed by zeaxanthin epoxidase (ZEP). Antheraxanthin is the intermediate product. All–*trans*-violaxanthin is converted to 9-*cis*-violaxanthin or 9′-*cis*-neoxanthin by the 9-*cis*-epoxy carotenoid dioxygenase (NCED), which yields a C15 intermediate product called xanthoxin. Then the product xanthoxin is exported to the cytosol (Nambara and Marion-Poll, 2005) where xanthoxin is converted to ABA. Xanthoxin is then converted into ABA by two enzymatic reactions. Finally, xanthoxin is converted to an ABA aldehyde by the enzyme, short-chain alcohol dehydrogenase/reductase (SDR), and then oxidation of the abscisic aldehyde to ABA is catalyzed by the abscisic aldehyde oxidase (AAO). (Modified from, Taylor et al., 2000; Finkelstein and Rock, 2002; Tuteja, 2007; Mehrotra et al., 2014).

Abscisic acid biosynthesis occurs in two places; it starts from plastids and ends in the cytosol. ABA in higher plants synthesized via the mevalonic acid-independent pathway also called indirect pathway. In this pathway, ABA is synthesized through cleavage of a C_40_ carotenoid precursor, followed by a two-step conversion of the intermediate xanthoxin to ABA via ABA aldehyde, which will be oxidized into ABA. Mutants defective in ABA biosynthesis have been isolated in many plant species from maize, tomato, tobacco, potato, barley and *Arabidopsis* (Xiong and Zhu, 2003). The primary mechanism of ABA biosynthesis pathway is shown in **Figure 1**.

The first step of ABA biosynthesis pathway is the conversion of zeaxanthin and antheraxanthin to all trans-violaxanthin, which will be catalyzed by zeaxanthin epoxidase (ZEP) in the plastid. The ZEP was identified by Marin et al. (1996). In this reaction, antheraxanthin is the intermediate formed. After that, all trans-violaxanthin converted to 9-*cis*-violaxanthin or 9′-*cis*-neoxanthin. The enzyme involved in this reaction is unknown (Seiler et al., 2011). After that, oxidative cleavage of 9-*cis*-violaxanthin and 9-*cis*-neoxanthin, catalyzed by the enzyme called 9-*cis*-epoxy carotenoid dioxygenase (NCED), which yields a C15 intermediate product called xanthoxin and C25 metabolite (Schwartz et al., 1997). *ZmNCED* gene was first isolated using the maize *viviparous14* mutant, and NCED is the key enzyme in ABA biosynthesis (Tan et al., 1997). Then the product xanthoxin is exported to the cytosol (Nambara and Marion-Poll, 2005) where xanthoxin is converted to ABA. In this step, xanthoxin is converted into ABA by two enzymatic reactions. First of all, xanthoxin is converted to an ABA aldehyde by an enzyme called short-chain alcohol dehydrogenase/reductase (SDR) encoded by the *AtABA2* gene in *Arabidopsis thaliana* (Rook et al., 2001; Cheng et al., 2002; Gonzalez-Guzman et al., 2002). The next and final step of ABA biosynthesis is oxidation of the abscisic aldehyde to ABA, catalyzed by the abscisic aldehyde oxidase (AAO).

### ABA Catabolism

When stress signals are diminished ABA is metabolized into inactive products (Ng et al., 2014). That is accomplished by two pathways called hydroxylation and conjugation (Nambara and Marion-Poll, 2005). In hydroxylation, ABA is hydroxylated via oxidation of three methyl groups (C-7′, C-8′, and C-9′) of the ring structure. Among these C-8′ is known as the dominant catalytic pathway (Zeevaart and Creelman, 1988; Cutler and Krochko, 1999; Okamoto et al., 2009). In the 8′-hydroxylation pathway, phaseic acid (PA) and dihydro phaseic acid (DPA) are the amplest ABA catabolites (Gillard and Walton, 1976; Kushiro et al., 2004; Saito et al., 2004; Nambara and Marion-Poll, 2005). Cytochrome P450 type enzyme (CYP707A) is a crucial enzyme for ABA metabolism (Kushiro et al., 2004; Saito et al., 2004). The metabolism is controlled by the expression of *CYP707A* gene in the stomata and vascular tissues of leaves under rehydrated conditions (Nambara and Marion-Poll, 2005; Okamoto et al., 2009, 2011). According to Lee et al. (2006), the activation of glucosidase via stress-induced polymerization also rapidly increases the active pools of ABA. Similarly, Okamoto et al. (2009) reported that CYP707A3 functions in vascular tissues to reduce systematic ABA levels. On the other hand, CYP707A1 catabolizes local ABA pools in guard cells in response to high humidity. The second pathway for inactivating ABA is conjugation. ABA conjugation plays a significant role in the regulation of the amounts under both normal and dehydration conditions (Lee et al., 2006; Xu et al., 2012). ABA and its hydroxylated catabolites can be conjugated to glucose. ABA glucosyl ester (ABA-GE) is synthesize by glycosyltransferase. ABA-GE is synthesized in the cytosol and stored in vacuoles (Lim et al., 2005; Boursiac et al., 2013). Under abiotic stress conditions, ABA glucosyl ester could be converted to ABA by enzyme-catalyzed hydrolysis. The glycosidase enzyme catalyzing the hydrolysis of ABA-GE to free ABA was first demonstrated in barley (Dietz et al., 2000). Subsequently, two ABA-GE hydrolyzing enzymes, BG1 (beta-glycosidase homolog 1) and BG2, have been isolated in *Arabidopsis* (Lee et al., 2006; Xu et al., 2012). BG1 is found in endoplasmic reticulum whereas BG2 is present in the vacuole (Lee et al., 2006; Xu et al., 2012). The ABA released from ABA-GE may not be required for fast stomatal responses, as the loss-of-function mutant of BG1 and BG2, *bg1bg2*, show wild-type like stomatal responses to reduced air humidity, elevated CO_2_, and ABA (Merilo et al., 2015).

### ABA Transport

The translocation of ABA between cells, tissues and organs also play important roles in whole plant physiological response to stress conditions. ABA, being a weak acid, can diffuse passively across biological membranes when it is protonated (Wilkinson and Davies, 2010; Ng et al., 2014). ABA can also be transported across membranes by transporters. ABA transporters were first identified in *Arabidopsis*, and they are ATP-binding cassette (ABC)-containing transporter proteins (Kang et al., 2010; Kuromori et al., 2010). *AtABCG25* encodes a half-size ABC transporter protein and which is responsible for exporting ABA from vascular tissues, the main sites of ABA synthesis in plants (Kuromori et al., 2010); AtBCG40, a full-size ABC transporter, acts as an ABA importer in plant cells (Kang et al., 2010). Subsequently, AtABCG22 was shown to be required for stomatal regulation and proposed to function as ABA transporter (Kuromori et al., 2011). Later, Ji et al. (2014) reported that ABCG16 is involved in ABA tolerance. Currently, Kang et al. (2015) have demonstrated four AtABCG protein functions together to supply ABA in mature imbibed seeds. They showed that AtABCG25 and AtABCG31 export ABA from the endosperm to the embryo, whereas AtABCG30 and AtABCG40, transport ABA into the embryo. These studies demonstrate that certain ATP-binding cassette (ABC)-containing transporter proteins are important for ABA transport and responses. In addition to ABA transporters belonging to the ABC family, other types of ABA transporters are also found. Kanno et al. (2012) identified an ABA-importing transporter (AIT1) by transport assay in yeast and insect cells. AIT1 belongs to the NRT1 (nitrate transporter 1) transporter family. *ait1* mutants were less sensitive to ABA during germination and post-germination growth and stomata remained open whereas overexpression of *AIT1* resulted in ABA hypersensitivity (Kanno et al., 2012). Recently, AtDTX50 (Detoxification Eﬄux Carrier 50) has been found to participate in ABA transport in *Arabidopsis* (Zhang et al., 2014). AtDTX50 is a membrane protein in the MATE (Multidrug and Toxic Compound Extrusion) transporter family (Zhang et al., 2014). The *atdtx50* mutant showed faster ABA-induced stomatal closure, indicating increased ABA accumulation in the guard cells that confer drought tolerance. *AtDTX50* is primarily expressed in vascular tissues and guard cells. These results indicate that AtDTX50 mediates ABA eﬄux from the cytosol of vascular and guard cells located in the plasma membrane (Zhang et al., 2014). It appears that multiple types of transporters are involved in ABA transport in plants.

## ABA Signaling

**FIGURE 2:**
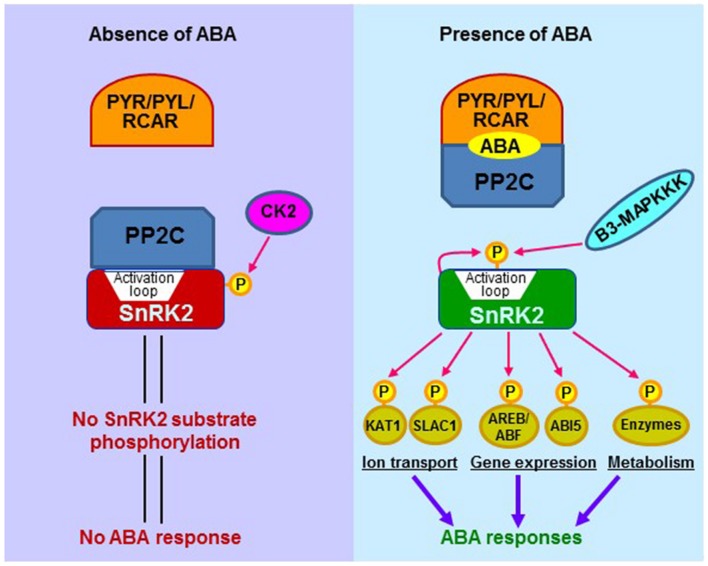
**The schematic representation of major ABA signaling pathway in plants with and without ABA presence.** The core components in ABA signaling include ABA receptors (PYR/PYL/RCAR), PP2C phosphatases (negative regulators), and SnRK2 kinases (positive regulators). In the absence of ABA, PP2Cs associate with SnRK2s and prevent the activation of SnRK2s. The inactive SnRK2s are unable to phosphorylate downstream substrates, and thus signal transduction is not occurring. In the presence of ABA, PYR/PYL/RCAR receptors bind to ABA and interact with PP2Cs, which release SnRK2s. The SnRK2s are then activated by autophosphorylation of the activation loop. The active SnRK2s can phosphorylate downstream substrate proteins, including transcription factors, ion channels, and enzymes such as NADPH oxidases, thereby inducing ABA responses. SnRK2s are subjected to regulation by other protein kinases. A Raf-like kinase (B3-MAPKKK) has been shown to activate SnRK2 through phosphorylating the activation loop, whereas casein kinase 2 (CK2) can phosphorylate SnRK2’s carboxyl-terminal serine residues, thereby enhancing SnRK2-PP2C interaction and inactivating SnRK2. Catalytically active SnRK2 is shown in green and inactive SnRK2 is in red. ABF, ABA-responsive element binding factor; ABI5, ABA insensitive 5; AREB, ABA-responsive element binding protein; B3-MAPKKK, B3-group Raf-like MAP kinase kinase kinase; KAT1, potassium channel in *Arabidopsis thaliana* 1; PP2C, Protein phosphatase 2C; PYR, pyrabactin resistance; PYL, PYR-related; RCAR, regulatory component of ABA receptor; SLAC1, slow anion channel 1; SnRK2, sucrose nonfermenting-1-related protein kinase 2.

Abscisic acid is a key endogenous messenger in plants, and it has a crucial role in various plant stresses. Therefore, understanding the signaling mechanism of ABA is critical for improving plant performance under stress environments and most needed in projected warmer and drier future climate. There are three core components of ABA signaling; pyrabactin resistance (PYR)/pyrabactin resistance-like (PYL)/regulatory component of ABA receptors (RCAR), protein phosphatase 2C (PP2C: acts as negative regulators) and (Sucrose non-fermenting) SNF1-related protein kinase 2 (SnRK2: acts as positive regulators). In the presence of ABA, PYR/PYL/RCAR-PP2C complex formation leads to inhibition of PP2C activity, which allows the activation of SnRK2. Activated SnRK2 then phosphorylates downstream substrate proteins such as transcription factors, and thus facilitating transcription of ABA-responsive genes (**Figure 2**).

### The PYR/PYL/RCAR ABA Receptors

Two independent research groups (Ma et al., 2009; Park et al., 2009) made remarkable discovery of an ABA receptor. They identified *Arabidopsis* PYR1 (pyrabactin resistance 1), PYL (PYR-related) or RCAR (Regulatory Component of ABA Receptor) proteins as ABA receptors. The *Arabidopsis* genome contains 14 PYR/PYL/RCAR genes, which encode highly conserved small proteins with 159-211 amino acid residues (Ma et al., 2009; Park et al., 2009; Nishimura et al., 2010). Many of PYR/PYL/RCARs including PYR1, PYL1 and PYL2 have been shown to directly bind ABA (Miyazono et al., 2009; Nishimura et al., 2009; Santiago et al., 2009; Yin et al., 2009). Once bound to ABA this receptor can bind to group A protein phosphatases (PP2C), including ABI1 (ABA-insensitive 1) and ABI2, the negative regulator of ABA signaling (Ma et al., 2009; Park et al., 2009; Nishimura et al., 2010). These ABA receptors played a critical role in ABA perception, as quadruple mutants *pyr1*, *pyl1*, *pyl2* and *pyl4* (recently six i.e., *pyr1/pyl1/pyl2/pyl4/pyl5/pyl8*) are insensitive toward ABA (Park et al., 2009; Gonzalez-Guzman et al., 2012). They also showed hyposensitivity in germination and root growth response to ABA (Park et al., 2009; Gonzalez-Guzman et al., 2012). Overexpression of PYL5, PYL8 or PYL9 enhanced ABA responses or conferred drought resistance to *Arabidopsis thaliana* (Ma et al., 2009; Santiago et al., 2009; Saavedra et al., 2010). The PYR/PYL/RCAR family in *Arabidopsis* (except PYL13) is capable of activating ABA signaling response, suggesting that nearly all members can function as ABA receptors (Fujii et al., 2009). Antoni et al. (2013) reported that ABA-dependent inhibition of PP2Cs by PYR/PYLs is essential for the perception of moisture gradient. The structural and molecular studies conducted by many scientists have convincingly shown that PYR/PYL/RCAR receptors play a central role in ABA perception (Ma et al., 2009; Miyazono et al., 2009; Nishimura et al., 2009; Park et al., 2009; Santiago et al., 2009; Melcher et al., 2010).

### PP2C: The Negative Regulators

Reversible protein phosphorylation catalyzed by protein kinases and phosphatases play a significant role in cellular signal transduction, which helps in the transmission of signals from external to the internal environment of the cell. There are 112 phosphatases encoded in the *Arabidopsis* genome, among them 76 genes encode for PP2Cs (Schweighofer et al., 2004). At least 6 of the nine members of the group A PP2Cs have been shown to involved in ABA signaling, among them, ABI1, ABI2, and HAB1 are the well characterized. The members of the group A have defined roles in different tissues with tissue-specific expression patterns (Ng et al., 2014). The PP2C group A genes first characterized are *ABI1* and *ABI2*, which was isolated from a genetic screen in *Arabidopsis*. Both *abi1* and *abi2* mutants show insensitivity in various tissues, developmental stages, suggesting that they act as a negative regulator of ABA signaling. Both of these mutants display reduced seedling growth, seed dormancy, drought tolerance and stomatal regulations (Leung et al., 1994, 1997; Meyer et al., 1994; Rodriguez et al., 1998; Saez et al., 2004; Schweighofer et al., 2004; Yoshida T. et al., 2006). Few years later, HAB1 (homolog to ABI1) and HAB2 were isolated based on their sequence similarity to ABI1 (Saez et al., 2004). Similarly, other members of A group PP2Cs such as AHG1 and AHG3/AtPP2CA were identified in genetic screens of *Arabidopsis* and yeast complementation test. It has been shown that all of the loss-of-function mutants of group A PP2Cs exhibit significant ABA hypersensitivity, which establishes them as the major negative regulators of ABA signaling (Kuromori and Yamamoto, 1994; Kuhn et al., 2006; Yoshida R. et al., 2006; Nishimura et al., 2007; Umezawa et al., 2010; Antoni et al., 2012). ABI1, ABI2, and HAB1 showed a dominant role in ABA signaling pathways in both seeds and vegetative tissues; the evidence is supported by gene expression data and genetic analysis. Recessive *hab1-1* mutants showed enhanced ABA-responsive gene expression, increased ABA-mediated stomatal closure and ABA-hypersensitivity in seed germination. These results indicate that HAB1 negatively regulates ABA signaling (Gosti et al., 1999; Saez et al., 2004; Hirayama and Shinozaki, 2007). These results are consistent with the fact that PP2Cs function as negative regulators of ABA signaling (Mehrotra et al., 2014). Recently, Singh et al. (2015) reported a group A PP2C from rice gene (*OsPP108*), whose expression is highly inducible under salt and drought stresses. The overexpression of *OsPP108* confers ABA insensitivity and tolerance to stresses like high salt and mannitol (Singh et al., 2015).

### SnRK2 Protein Kinases: The Positive Regulators

The identification of PP2Cs showed that reversible protein phosphorylation process is primary in ABA signaling pathway. Protein kinases are known to be involved in ABA signaling pathway. The sucrose non-fermenting 1 (SNF1)-related protein kinase2 (SnRK2) family of protein kinases are plant specific serine/threonine kinases participating in plant response and has a central role in cellular responses to drought and dehydration (Hrabak et al., 2003; Saruhashi et al., 2015). The first protein kinase having ABA-stimulated catalytic activity was identified as an AAPK in *Vicia faba* (Li and Assmann, 1996; Li et al., 2000). AAPK is a positive regulator of ABA-induced stomatal closure (Li et al., 2000). Subsequently, the AAPK ortholog in *Arabidopsis*, OST1 (Open Stomata 1)/SnRK2.6, was shown to function in ABA-mediated stomatal regulation (Mustilli et al., 2002; Yoshida et al., 2002). Similar to AAPK, ABA-activated protein kinases have been identified in *Arabidopsis* (Boudsocq et al., 2004) and rice (Kobayashi et al., 2004); they belong to the SnRK2 family. These ABA-activated protein kinases mediate ABA signaling by phosphorylating their substrate proteins such as transcription factors, ion channels, and metabolic enzymes (Umezawa et al., 2010; Fujita et al., 2013). According to Gómez-Cadenas et al. (1999), transcription of PKABA1 is induced by ABA and occurs in embryos and seedlings. The function of PKABA1 is to mediate ABA-suppressed gene expression. PKABA1 phosphorylates and activates the ABA-responsive basic-domain leucine-zipper (bZIP) transcription factor TaABF, which help in regulation of ABA signaling in seed development and germination (Shen et al., 2001; Johnson et al., 2002). Similarly, AAPK modulates an RNA-binding protein AKIP1 by phosphorylating it and inducing its translocation into subnuclear speckles in guard cells (Li et al., 2000, 2002).

SnRK2 family is the key regulator of plant response to abiotic stress and is divided into three sub-groups depending upon the affinity toward ABA. Subgroup-I kinases do not respond to ABA whereas subgroup-III actively respond to ABA and believed to be the key regulator of ABA-dependent pathway gene expression. Subgroup-II weakly responds or did not provide a response to ABA (Hrabak et al., 2003; Boudsocq et al., 2004; Kulik et al., 2011). There are altogether 10 SnRK2 members found in *Arabidopsis thaliana*, i.e., SnRK2.1-SnRK2.10 or SnRK2A-SnRK2J. Among them, 5 SnRK2 members (SnRK2.2., SnRK2.3, SnRK2.6, SnRK2.7, and SnRK2.8) can be activated by ABA. The all of the SnRK2s except SnRK2.9 can be activated by osmotic stress, which indicated that most SnRK2 members are involved in ABA and stress signaling (Boudsocq et al., 2004; Furihata et al., 2006; Yoshida T. et al., 2006). Among them, SnRK2.2, SnRK3.3, and SnRK2.6 (Subgroup-III) are known as primary regulators of ABA since they exhibit the strongest activation of ABA (Boudsocq et al., 2004). The first member of the *Arabidopsis* SnRK2 family is OST1/SnRK2.6 which identified by forward genetic approach and act as a regulator of ABA signaling. The mutant *snrk2.6* (*ost1*) cannot exhibit ABA-induced stomatal closure, indicating that it positively regulates the stomatal response to ABA. The *SnRK2.6* gene mainly expressed in guard cells, and the vascular system whereas seed germination and post-germination growth were not affected in *snrk2.6* loss-of-function mutant, which indicated that the SnRK2.6 protein kinase may not function in seed germination (Mustilli et al., 2002; Yoshida et al., 2002). Moreover, SnRK2.2 and SnRK2.3 function redundantly in ABA-induced inhibition of seed germination and post-germination growth (Fujii et al., 2007). According to Fujii et al. (2007), *snrk2.2* and *2.3* single mutants show weak, while *snrk2.2* and *snrk2.3* double mutant exhibits high ABA-insensitive phenotypes regarding seed germination and seedling growth. The triple mutation (*snrk2.2/2.3/2.6*), nearly block all the main ABA responses (Fujii and Zhu, 2009; Fujita et al., 2009; Nakashima et al., 2009a; Waadt et al., 2015). In *Arabidopsis* proteins extract, these 3 SnRK2s, along with 9 of the 14 members of PYR/PYL/RCARs, could be co-immunoprecipitated with ABI. Though the composition of co-purified proteins was ABA-independent, these data suggest that at least ABI1, the 3 SnRK2s and at least 9 of the 14 receptor proteins may constitute a core signaling complex (Fujii and Zhu, 2009; Nishimura et al., 2010; Joshi-Saha et al., 2011). The expression profile of these three SnRK2s genes supports their redundant, supportive roles in the ABA responses (Fujii et al., 2007, 2011). Double mutant *snrk2.7/2.8* affect the expression of ABA and drought responsive genes, indicating that SnRK2.7/2.8 has potential roles in ABA signaling although *snrk2.7* and *snrk2.8* single mutation show wild-type phenotypes due to functional redundancy (Mizoguchi et al., 2010).

Many studies reported that phosphorylation at the activation loop is critical for the activation of SnRK2s. Several phosphorylation sites have been identified in SnRK2.6; ABA stimulates phosphorylation of Ser 175 in the activation loop, whereas PP2C can dephosphorylate the Ser175 of SnRK2.6 in the absence of ABA, resulting in the deactivation of SnRK2.6 (Fujii et al., 2009; Ma et al., 2009; Park et al., 2009; Umezawa et al., 2009; Vlad et al., 2010). Quantitative analysis confirmed that ABA treatment increases the phosphorylation of the site *in vivo* (Kline et al., 2010) and phosphorylation of the site is also increased by osmotic stress (Vlad et al., 2010). SnRK2 activation loop phosphorylation may also implicate unidentified upstream kinases in plants (Burza et al., 2006; Boudsocq et al., 2007). ABA- or stress-activated SnRK2 family members have been found in *Vicia faba*, soybean, *Arabidopsis*, tobacco, rice, *Chlamydomonas reinhardtii*, maize, sorghum, and wheat (Li et al., 2000; Monks et al., 2001; Boudsocq et al., 2004; Kelner et al., 2004; Kobayashi et al., 2004; Gonzalez-Ballester et al., 2008; Huai et al., 2008; Li et al., 2010; Mao et al., 2010; Zhang et al., 2010b).

Understanding how SnRK2 kinases exert their effects in ABA signaling network requires knowledge of SnRK2 targets. Several phosphorylation substrates of SnRK2 kinases have been identified using biochemical and molecular approaches. The ABFs (ABA-responsive-element Binding Factor)/AREBs (ABA-Responsive Element Binding factors) transcription factors involving in regulating ABA-responsive genes are SnRK2 substrates as ABA-activated SnRK2 kinases have been shown to phosphorylate ABF/AREB proteins *in vitro* and *in vivo* (Kobayashi et al., 2005; Furihata et al., 2006; Fujii et al., 2007; Sirichandra et al., 2010). Another transcription factor involved in ABA signaling, ABI5, can be phosphorylated *in vitro* by ABA-activated SnRK2 kinases (Fujii et al., 2007; Nakashima et al., 2009a; Wang et al., 2013b). In addition to nuclear transcription factors, SnRK2 can also phosphorylate plasma membrane proteins. The guard cell OST1/SnRK2.6 has been shown to phosphorylate the anion channel SLAC1, the potassium channel KAT1, and the NADPH oxidase AtrbohF, all of which are plasma-membrane proteins important in controlling stomatal aperture (Geiger et al., 2009; Lee et al., 2009; Sato et al., 2009; Sirichandra et al., 2009). To identify more potential SnRK2 targets, phosphoproteomic approaches have been used to survey ABA-responsive phosphoproteins. Six ABA-regulated phosphoproteins (ascorbate peroxidase, Ca^2+^/H^+^ antiporter regulator protein, G protein beta subunit-like protein, glyoxysomal malate dehydrogenase, manganese superoxide dismutase, and triosephosphate isomerase) have been identified from rice leaves by using 2-D gel electrophoresis, phospho-antibody immunoblotting, and mass spectrometry (He and Li, 2008). Using *snrk2.2/2.3/2.6* triple knockout mutant and comparative phosphoproteomics, two research groups have identified respectively 35 and 58 putative SnRK2 substrates (Umezawa et al., 2013; Wang et al., 2013a). The putative SnRK2 substrates identified by the two groups show little overlap, suggesting that the three ABA-activated protein kinases (SnKR2.2, SnKR2.3, and SnKR2.6) can target and regulate a range of proteins via ABA-mediated phosphorylation.

### Possible Regulator of SnRK2

Recently, a novel regulatory component ARK is identified by Saruhashi et al. (2015). They reported that ARK was an essential signaling component for the regulation of SnRK2 in basal land plants. A single gene encodes ARK (ABA, and abiotic stress-responsive Raf-like kinase) in moss (*Physcomitrella patens*) which belongs to the family of group B3 Raf-like MAP kinase kinases (B3-MAPKKKs). Phosphorylation and activation events in these kinases are modulated through their non-kinase domain by dimerization, binding of other regulatory proteins or phosphorylation by upstream MAPKKK kinases (Kolch, 2000). However, Saruhashi et al. (2015) reported that ARK might be activated itself by autophosphorylation in the activation loop by using transient assay of phosphopeptide mapping and mutational analysis. Their study showed that ARK provides positive regulation of ABA signaling in addition to the negative relation by group A PP2Cs in bryophytes, which provides new insights into a signaling pathway for a possible connection between these kinases through unknown mechanisms operating in plant ABA response. They also revealed that hyperosmosis induced activation of plant SnRK2s mediated by an upstream of ARK (Saruhashi et al., 2015). Recently the other study also reported that hyperosmosis mediated response by both ABA-dependent and independent mechanism using ABA-deficient mutant of *P. patens* (Takezawa et al., 2015). To understand the molecular mechanism, they identified one mutant AR7 (ARK) and microarray profiles showed that the expression of a majority of the ABA-responsive genes was affected in AR7. The expression of 518 out of 579 ABA upregulated genes in the wild-type line was reduced significantly in AR7. These genes are included 27 LEA-like genes which are responsible for dehydration stress tolerance. RNA gel blot analysis also confirmed impaired ABA-induced gene expression in AR7 (Saruhashi et al., 2015). Furthermore, levels of 150 of 165 ABA down-regulated transcripts were higher in AR7 than in wild type. They showed that ARK could phosphorylate serine residues in the activation loop of SnRK2B thus activating SnRK2B and ABA signaling (Saruhashi et al., 2015). These results indicated that gene impaired in AR7 encodes an important positive regulator of ABA-responsive gene expression in *P. patens.* All these findings suggested that B3-MAPKKKs in angiosperms are involved in positive regulation of SnRK2 in ABA response and SnRK2 activation.

In contrast to ARK, another new negative regulator of SnRK2 has been reported recently Vilela et al. (2015) identified Casein Kinase 2 (CK2) as a negative regulator of SnRK2 in maize. CK2 is an evolutionary multi-subunit serine/threonine kinase and found in all eukaryotes (Meggio and Pinna, 2003; Vilela et al., 2015). Among all kinase, CK2 is unique because it can use either ATP or GTP as phosphoryl donors (Niefind et al., 1999). CK2 is involved in plant growth and development, light-regulated gene expression, hormone responses, cell-cycle regulation, flowering time, DNA repair or responses to abiotic stress and biotic stresses in plants (Lee et al., 1999; Riera et al., 2004; Portolés and Más, 2007; Moreno-Romero et al., 2008; Riera et al., 2013; Mulekar and Huq, 2014; Vilela et al., 2015). In the case of abiotic stresses, CK2 acts as an ABA regulator because *ck2α* mutants are hypersensitive to ABA concerning seed germination, cotyledon greening, and stomatal opening (Mulekar et al., 2012; Wang et al., 2014). These effects were attributed to the down-regulation of ABA-related genes, including OST1, but the biochemical relationship between CK2 and OST1 was not clear. In *Arabidopsis*, OST1 is the best genetically and biochemically characterized SnRK2, functioning as a key regulator in the core of ABA signaling module (Cutler et al., 2010). CK2 can phosphorylate the ABA box of ZmOST1 to accelerate the latter’s turnover and to promote binding to PP2Cs, suggesting that CK2 is a regulator of OST1 protein stability (Vilela et al., 2015). They also showed that overexpression of *ZmOST1* in transgenic *Arabidopsis* plants exhibit highly resistant to drought and hypersensitive to ABA at the level of stomata. These two newly discovered SnRK2 regulators (B3-MAPKKK and CK2) will add more focused research toward ABA signaling pathway (**Figure 2**).

## ABA Signaling Response at Gene Expression Levels

### Transcription Factors Involved in ABA-Regulated Gene Networks

Transcription factors are master regulators that integrate, balance, and coordinate hormonal, developmental and environmental signals in plant systems (Jaradat et al., 2013). A single transcription factor can regulate the expression of many target genes through specific binding of a transcription factor to *cis*-acting elements in promoters of respective target genes. Plant genomes assign approximately 7% of their coding sequences to transcription factors, which provides the complexity of transcriptional regulation (Udvardi et al., 2007). Transcription factors significant for the regulation of ABA-related gene network include AREBs (ABA-responsive element binding proteins)/ABFs (ABRE binding factors), ABI5 (ABA insensitive 5), MYB (myeloblastosis), MYC (myelocytomatosis), NAC (NAM: no apical meristem; ATAF: *Arabidopsis* transcription activation factor; CUC: cup-shaped cotyledon), and ERF (ethylene response factor).

AREBs/ABFs belong to the bZIP (basic leucine zipper) transcription factor subfamily. They were isolated based on their interaction with ABRE (ABA-responsive element) *in vitro* and regulatory function in ABA and or stress responses (Choi et al., 2000; Uno et al., 2000). For example, AREB1/ABF2, AREB2/ABF4, and ABF3 can be induced by high salt, dehydration or ABA treatment in vegetative tissues (Riechmann et al., 2000; Yoshida et al., 2010). Kim et al. (2004) reported that overexpression of ABF3 confers tolerance to chilling, freezing, high temperature, and drought stress. Similarly, ABP9 (ABA-responsive-element binding protein 9) is also a member of the bZIP family found to be associated with drought and heat stress. Constitutively expression of ABP9 in *Arabidopsis* improve photosynthetic capacity under water deficit and heat shocks, suggesting that ABP9 has a significant role in the regulation of plant photosynthesis under stress conditions (Zhang et al., 2008b).

ABI5 is also a member of *Arabidopsis* bZIP transcription factor subfamily and has four conserved domains (Jakoby et al., 2002). The ABI5 expression is higher in mature seeds and young seedling exposed to ABA or dehydration stress (Finkelstein and Lynch, 2000; Lopez-Molina et al., 2001). ABI5 can bind to ABRE in the promoters of its target genes and is a positive regulator of ABA-regulated transcription networks (Shen et al., 1996; Finkelstein and Lynch, 2000). Similar to *Arabidopsis* ABI5, overexpression and upregulation of OsABI5 in rice under ABA and salinity conditions confers salt tolerance (Zou et al., 2008).

MYC and MYB, belonging to the bHLH (basic helix loop helix) subfamily, also play a regulatory role in ABA signaling by activating some stress-inducible genes (Abe et al., 2003). MYB in *Arabidopsis* namely AtMYB60, AtMYB44, and AtMYB15 have been shown to be involved in the regulation of stomatal closure and ABA-mediated response to drought and salt stresses (Jaradat et al., 2013). The *Arabidopsis* MYC transcription factor, AtMYC2, is an ABA-responsive gene induced by drought and salt (Abe et al., 1997).

NAC transcription factors appear to be involved in ABA transcriptional network. The *Arabidopsis* NAC-type transcription factor, AtNAC, has been shown to play a fundamental role in the regulation of ABA- and senescence-induced genes (Zhang and Gan, 2012). Furthermore, ABA can induce the expression of seven stress-induced NAC transcription factors in *Arabidopsis* (Takasaki et al., 2015). Loss-of-function mutation of these NAC transcription factor genes confers retardation of leaf senescence induced by ABA, suggesting that NAC transcription factors play a key role ABA-induced leaf senescence. Recently, Jiang et al. (2014) reported that the *RhNAC3* transcription factor is involved in regulating ABA signaling pathway in *Arabidopsis* and rose. Overexpression of *RhNAC3* in *Arabidopsis* resulted in ABA hypersensitivity during seed germination and enhanced stomatal closure in response to ABA or drought treatments. Furthermore, the expression of downstream genes of ABA signaling pathways was repressed in *RhNAC3*-silenced petals in a rose, suggesting that the RhNAC3 transcription factor plays a role in regulating ABA-related genes.

Finally, ERF-type transcription factors have been implicated in regulating ABA response. Overexpression of *AtERF7* (*Arabidopsis* ERF 7) caused reduced sensitivity to ABA in guard cells and increased transpiration water loss, whereas repression of AtERF7 via RNA interference resulted in increased sensitivity to ABA (Song et al., 2005). These findings show that the ERF7 transcription factor can play a significant role in regulating ABA responses. Similarly, a tomato ERF-type transcription factor, JERF3, was shown to be transcriptionally induced by salt and ABA and overexpression of JERF3 leads to tobacco plants with enhanced tolerance to salinity (Li et al., 2008). Therefore, the transcription factors mentioned in the above sections illustrate the regulation of the complex ABA-dependent transcription networks in plants under stress conditions.

### ABA-Induced Gene Expression by Activation of SnRK2

Drought and salt stress-induced the accumulation of ABA, which then act as a stress signal (Ng et al., 2014). According to Cutler et al. (2010), ABA signaling leads to substantial changes in gene expression, which implicate changes in transcription, transcript processing, and stability. Many studies reported that almost 10% of the *Arabidopsis* genes are regulated by ABA (Hoth et al., 2002; Nemhauser et al., 2006; Yang et al., 2008). In the other study, Böhmer and Schroeder (2011) identified 3494 ABA-responsive transcripts and 50 ABA-responsive proteins using microarrays and quantitative proteomics. Among the identified ABA-responsive genes, 1512 genes were upregulated, and 1982 genes were downregulated by ABA treatment.

Abscisic acid-dependent gene expression requires the binding of transcription factors to cis-regulatory elements in target genes promoters. The major cis-regulatory element associated with expression of ABA-responsive-genes is the ABA-response element (ABRE: PyACGTGG/TC), which may require an ABRE-coupling element (CE) for full function (Hobo et al., 1999; Zhang et al., 2005; Nakashima et al., 2014). ABRE-binding proteins (AREBs) or ABRE-binding factors (ABFs) are the transcription factors that can bind to the ABREs of ABA-responsive genes and activate their gene expression (Choi et al., 2000; Uno et al., 2000). Further, ABA-dependent phosphorylation of AREBs/ABFs in multiple RXXS/T sites is necessary for their activation (Uno et al., 2000; Kagaya et al., 2002). ABA-activated SnRK2 kinases have been shown to be responsible for ABA-dependent phosphorylation of AREBs/ABFs (Johnson et al., 2002; Kobayashi et al., 2005; Furihata et al., 2006; Fujii et al., 2007). In *Arabidopsis*, ABA-dependent upregulation of gene expression could be stamped out in the mutant lacking all three ABA-activated SnRK2 kinases (Fujii and Zhu, 2009; Nakashima et al., 2009a), suggesting that ABA-activated SnRK2s play an essential role in regulating the expression of ABA-responsive genes through phosphorylating AREBs/ABFs. Moreover, comparative transcriptome analyzes reveal that the majority but not all of ABA-responsive genes down-regulated in the triple mutant of ABA-activated SnRK2s are also down-regulated in the quadruple mutant of ABREs/ABFs (Yoshida et al., 2015). These results indicate that AREB1, AREB2, and ABF3 and ABF1 are the major transcription factors downstream of the ABA-activated SnRK2s in ABA signal transduction during vegetative growth. Nevertheless, these results also suggest that other transcription factors targeted by ABA-activated SnRK2s may contribute to transcriptional regulation of ABA-responsive genes.

ABA insensitive 5 (ABI5) is an important regulator of ABA responses during seed germination and seedling growth (Finkelstein and Lynch, 2000; Brocard et al., 2002). Like AREBs/ABFs, the basic leucine zipper (bZIP)-type transcription factor ABI can also bind to ABREs and regulate the expression of some ABA-responsive genes (Lopez-Molina et al., 2001; Kim et al., 2002; Yang et al., 2011). ABA-activated SnRKs can phosphorylate ABI5 (Nakashima et al., 2009a; Wang et al., 2013b) and phosphorylation of ABI5 is required for activating the transcription factor (Wang et al., 2013b). Dephosphorylation of ABI5 by serine/threonine protein phosphatase 6 can negatively regulate ABA signaling (Dai et al., 2013). These results indicate that ABA could regulate some ABRE-dependent gene expression via SnRK2-mediated phosphorylation and activation of ABI5 family transcription factors during cellular dehydration in seeds (Fujita et al., 2011).

The RAV1 (Related to ABI3/VP1) transcription factor, which belongs to the Ethylene Responsive Factor (ERF) family (Feng et al., 2005), is another transcriptional regulator targeted by the ABA-activated SnRK2s in *Arabidopsis* (Feng et al., 2014). Feng et al. (2014) demonstrated that RAV1 is a negative regulator of ABA signaling, and it suppresses the expression of ABA-responsive genes such as *ABI3, ABI4, ABI5, Em1*, and *Em6*. Biochemical analysis showed that the ABA-activated SnRK2s (SnRK2.2, SnRK2.3, and SnRK2.6) interact with and phosphorylates the RAV1 protein (Feng et al., 2014). Genetic analysis revealed that the ABA-activated SnRK2s could repress the activity of RAV1. Taken together, this work shows that ABA-activated SnRK2s can inactivate a transcription factor via phosphorylating it. Another study has shown that the ABA-activated SnRK2.6 is involved in phosphorylating and inactivating basic helix-loop-helix (bHLH)-type transcription factors, which have critical roles in stomatal regulation (Takahashi et al., 2013). It is interesting to see whether SnRK2s mediated phosphorylation of transcriptional regulators would contribute to regulating ABA-repressive genes. Earlier studies demonstrate that bZIP transcription factors (ABF1, ABF2/AREB1, and ABI5) play a positive role in ABA signaling and the transcriptional activity of these factors are activated via SnRK2s-mediated phosphorylation (Fujii et al., 2007; Fujii and Zhu, 2009; Nakashima et al., 2009b; Wang et al., 2013b). The work by Feng et al. (2014) showed that RAV1 is a negative regulator of ABA signaling and SnRK2s-mediated phosphorylation repress the activity of RAV1. Thus, SnRK2s, as core components in ABA signaling, can phosphorylate both positive and negative regulators in ABA signaling network. Not all ABA-responsive genes contain ABRE(s) in their promoter regions (Wang et al., 2011a; Kim, 2014). Therefore, transcription factors recognizing non-ABRE regulatory elements such as NAC and MYC/MYB may be involved in regulating these ABA-responsive genes. It remains to see if the ABA-activated SnRK2s have a role in the regulation of the activity of these transcription factors.

### Post-transcriptional Control in Regulating ABA Response

RNA-binding proteins, which interact directly with RNA molecules, have crucial roles in many aspects of post-transcriptional control of gene expression, including RNA splicing and processing, mRNA stabilization, mRNA localization and translation (Glisovic et al., 2008).

RNA-binding proteins typically contain one or more RNA-binding domains which include RNA-recognition motif, K homology domain, zinc finger, cold-shock domain, aspartate-glutamate-alanine-aspartate (DEAD) box, and double-stranded RNA-binding domain (Ambrosone et al., 2012). The current understanding suggests that RNA-binding protein-mediated post-transcriptional gene regulation plays a significant role in ABA response. The mutation in the *hyponastic leaves 1* (*HYL1*) gene encoding a double-stranded RNA-binding protein causes hypersensitivity to ABA during seed germination in *Arabidopsis* (Lu and Fedoroff, 2000). Similarly, the mutation in the *supersensitive to ABA and drought 1* (*SAD1*) gene encoding a polypeptide similar to Sm-like small nuclear ribonucleoproteins (snRNP) involving nuclear mRNA processing increases plant sensitivity to ABA and drought stress (Xiong et al., 2001). *Arabidopsis* mutant defective in the mRNA cap-binding protein, ABH1 (ABA hypersensitive 1), shows ABA-hypersensitive regulation of seed germination and stomatal closing (Hugouvieux et al., 2001, 2002). Mutation in the DEAD box RNA helicase gene *LOS4* (*low expression of osmotically responsive genes 4*) in *Arabidopsis* confers ABA hypersensitive and cold tolerance phenotype (Gong et al., 2005). The accumulation of poly (A)^+^ RNA in the nuclei in this mutant suggests that this RNA helicase plays a crucial role in mRNA export from the nucleus (Gong et al., 2005). Two other *Arabidopsis* DEAD-box RNA helicase genes have been shown to be down-regulated by ABA and multiple abiotic stresses (Kant et al., 2007). Insertional mutants of the two genes exhibit improved tolerance to salt, osmotic, and heat stresses, suggesting that these two DEAD-box RNA helicases are involved in regulating abiotic stress responses (Kant et al., 2007). In maize, a DEAD box RNA helicase (ZmDRH1) has been shown to interact with a glycine-rich RNA-binding protein (MA16), which can be induced by ABA and dehydration although their functions in ABA and stress responses are not understood (Gomez et al., 1988; Gendra et al., 2004). Another example of the involvement of RNA-binding proteins in ABA response in crop plants is ABA-activated protein kinase (AAPK)-interacting protein 1 (AKIP1), a heterogeneous nuclear ribonucleoprotein (hnRNP) in *Vicia faba* (Li et al., 2000, 2002). ABA treatment induces phosphorylation of AKIP1 and its relocation into the subnuclear structures resembling splicing speckles (Li et al., 2002). Similarly to AKIP1, its homolog in *Arabidopsis* called UBA2a [poly(U)-Binding Associated 2a] has also been shown to be translocated into nuclear speckles in response to ABA treatment (Riera et al., 2006). However, it is not certain whether the AKIP1 and UBA2a relocate into the genuine splicing speckles upon ABA treatment, the functional significance of nuclear re-localization of AKIP1 and UBA2a remain unclear (Lorković, 2009).

Besides hnRNPs, another class of RNA-binding proteins playing a key role in regulating alternative splicing is serine/arginine-rich (SR) proteins (Long and Caceres, 2009). Several studies have provided a link between plant SR proteins and ABA response. Two *Arabidopsis* SR genes (SR1 and SR33) have been shown to change their alternative splicing patterns in response to ABA treatment (Palusa et al., 2007). Also, gene expression analysis has identified six SR genes and the two SR-like genes from *Arabidopsis* to be involved in ABA-related responses (Cruz et al., 2014). Furthermore, a genetic study has pinpointed that the *Arabidopsis* SR45 protein as a negative regulator of glucose and ABA signaling in early seedling development (Carvalho et al., 2010). These studies indicate that post-transcriptional control of gene expression at the stages of pre-mRNA splicing and processing, mRNA stabilization, and mRNA export from the nucleus to the cytoplasm by RNA-binding proteins is critical for ABA signaling. Further identification of the direct targets of these RNA-binding proteins will be necessary to fully understand the molecular mechanisms underlying ABA action.

Besides post-transcriptional regulation, post-translational control of targeted proteins by ubiquitination, the covalent ligation of proteins to the highly conserved small protein called ubiquitin, has been shown to be involved in ABA signaling (Yu et al., 2016). Ubiquitination is carried out by three types of enzymes: ubiquitin-activating enzymes (E1s), ubiquitin conjugating enzymes (E2s), and ubiquitin ligases (E3s). Ubiquitination can affect proteins in different ways: it can tag proteins for degradation via the proteasome, change their cellular location, and promote or prevent interactions of proteins (Komander and Rape, 2012). Several key proteins in ABA signaling have been shown to be targeted by ubiquitination for proteasomal degradation. Low ABA levels enable recognition and ubiquitination of ABA receptors (e.g., PYL8) by the ubiquitin ligase substrate adaptor DDA1 and associated ubiquitin ligases (Irigoyen et al., 2014). Proteasomal degradation of ubiquitinated ABA receptors allows the release of PP2C phosphatases, negatively regulating SnRK2-mediated ABA signaling. On the other hand, increased ABA levels can stabilize the ABA receptors by restricting their ubiquitination (Irigoyen et al., 2014). Furthermore, the ubiquitin ligase RSL1 can target PYL4 and PYR1 ABA receptors in the plasma membrane and promote ubiquitination-mediated degradation of the ABA receptors (Bueso et al., 2014). The PP2C phosphatase ABI1 can also be ubiquitinated when it is associated the ABA receptor (e.g., PYR1). Proteasomal degradation of ABI1 may allow ABA signal to be transmitted more efficiently under stress conditions by eliminating the negative regulator of ABA signaling (Kong et al., 2015). Also, ABI5, a key regulator of ABA signaling, can be targeted by ubiquitination for proteasomal degradation, which could be important for inhibiting the expression of ABA-responsive genes regulated by ABI5 (Liu and Stone, 2013; Seo et al., 2014). Similarly, the abundance of the ABF1 and ABF3 transcription factor proteins is regulated by ubiquitination-mediated degradation, and ABA can stabilize ABF1 and ABF3 proteins (Chen et al., 2013). Two dozen ubiquitin ligases are known to participate in ABA signaling (Yu et al., 2016). For example, the endoplasmic reticulum-localized ubiquitin ligase SDIR1 (Salt-and Drought-Induced Really interesting new gene finger1) regulates ABA-mediated seed germination and salt stress response through ubiquitinating its substrate, SDIRIP1 (SDIR1-Interacting Protein 1) and subsequent proteasomal degradation (Zhang et al., 2015). The substrate proteins of some ubiquitin ligases implicated in ABA signaling are still not known. For instance, the ubiquitin ligase CHYR1 (CHY zinc-finger and Ring protein 1) promotes ABA-induced stomatal closure and improves plant drought tolerance (Ding et al., 2015). However, the substrate of CHYR1 is unknown. Nevertheless, substantial studies have demonstrated the important role of ubiquitination in many steps of ABA signal transduction (Yu et al., 2016).

### Epigenetic Regulation of ABA Response

Epigenetics are the changes in gene function that are not due to changes in DNA nucleotide sequence, but rather due to chemical modification of DNA and its associated proteins (Bonasio et al., 2010). Epigenetic modifications comprise DNA methylation, histone modifications, and the production of microRNA (Yaish et al., 2011). As environmental stress factors can cause epigenetic modification of the genome, which provides an important mechanism for mediating gene-environment interplay (King, 2015). Recent studies suggest that epigenetic modification plays a significant role in plant response to abiotic stresses such as drought, salinity, heat, and cold (Kim et al., 2015). Here, we provide insights on epigenetic regulation of ABA-related stress signaling networks.

DNA methylation is the addition of a methyl group to the cytosine residues in DNA by DNA methyltransferases. In plants, cytosines in the contexts of CG, CHG, and CHH (H = A, C, or T) can be methylated (Law and Jacobsen, 2010). While CG methylation can occur in active genes, CHG and CHH methylation is almost exclusively present in heterochromatin (Stroud et al., 2013). DNA methylation in promoter regions is accompanying with repression of gene expression. The effect of DNA methylation in the gene body varies according to the level of methylation: extreme low or high level of DNA methylation correlates with lower gene expression while modest DNA methylation is associated with higher gene expression (Zemach et al., 2010). Two recent studies suggested that ABA can regulate gene expression through DNA methylation. Khraiwesh et al. (2010) showed that ABA represses gene expression in *P. patens* via DNA methylation of gene promoters. In another study, ABA was shown to increase DNA methylation of promoters in three ABA-repressive genes in *Arabidopsis* crown galls, indicating the importance of DNA methylation in gene regulation by ABA (Gohlke et al., 2013). Further studies are needed to elucidate more ABA-responsive genes for DNA methylation changes in response to ABA to understand the role of ABA in DNA methylation-dependent gene regulation.

Among histone modifications, histone acetylation has been shown to be important in regulating ABA-responsive genes and stress responses. Overexpression of AtHD2C (an *Arabidopsis* histone deacetylase gene) in *Arabidopsis* resulting in enhanced expression of ABA-responsive genes and improved drought tolerance (Sridha and Wu, 2006) suggest that histone deacetylation could play a fundamental role in activating ABA-responsive genes in acclimating plants to drought. Conversely, mutation or repression of a histone deacetylase gene HDA6 causing decreased expression of ABA and abiotic stress-responsive genes confers ABA and salt stress-hypersensitive phenotypes (Chen et al., 2010). Similarly, HDA19 histone deacetylase mutants also showed hypersensitive to ABA and salt stress (Chen and Wu, 2010). Furthermore, mutation of the HOS15 gene, which encodes a protein similar a component in the human nucleosome deacetylation complex, confers ABA and salt stress-hypersensitive phenotypes (Zhu et al., 2008; Chinnusamy et al., 2010). These studies indicate that histone acetylation and deacetylation play a critical role in the regulation of ABA-responsive genes and plant responses to abiotic stresses. In addition, chromatins remodeling factors and nucleosome assembly proteins have also been implicated in ABA responses. The chromatin remodeling protein BRM in *Arabidopsis* has been shown to repress ABA responses by remodeling the nucleosomes at the *ABI5* (ABA insensitive 5) locus and thus inactivating expression of the ABA-related transcription factor *ABI5* (Han et al., 2012). Three nucleosome assembly proteins in *Arabidopsis* (AtNAP1;1, AtNAP1;2, and AtNAP1; 3) have been reported to function as positive regulators of ABA signaling responses (Liu et al., 2009a).

MicroRNAs (miRNAs) are short non-coding RNA that regulates gene expression post-transcriptionally by complementing the target mRNAs causing target mRNA degradation or translational repression (Bartel, 2009). miRNAs can exert epigenetic regulation of gene expression by downregulation of key epigenetic regulators such as DNA methyltransferases and histone deacetylases (Sato et al., 2011). miRNA genes with changed expression in response to ABA have been identified in plants. In *Arabidopsis*, ABA upregulates the expression of miR159, miR393, and miR402, whereas miR169a can be downregulated by ABA (Sunkar and Zhu, 2004; Reyes and Chua, 2007; Li et al., 2008). The predicted target of miR402 is the transcript of Demeter-like protein 3 (DML3), a DNA glycosylase involved in DNA demethylation. It has been hypothesized that miR402 under ABA induction reduces the transcript level of DML3 resulting in repression of DML3-targeted genes (Sunkar and Zhu, 2004; Chinnusamy et al., 2008). ABA-induced miR159 accumulation can cause MYB33 and MYB101 transcript degradation, which is believed to desensitize ABA signaling during stress responses (Reyes and Chua, 2007). miR169a targets the transcript of NFYA5, a transcription factor vital in regulating plant development and stress responses. Downregulation of miR169 by ABA can contribute to lead to increased expression of NFYA5 and promote drought resistance (Li et al., 2008). In rice, ABA downregulates miR167 expression (Liu et al., 2009b). Interestingly, drought treatment can decrease miR167 expression and increase the expression of PLD (phospholipase D), which is the potential target of miR167 (Wei et al., 2009). As PLD is an important regulator of ABA response and stress signaling in plants (Guo and Wang, 2012), miR167 is likely to be involved in regulating ABA-dependent stress signaling networks. Global expression profiling in rice has identified 34 miRNAs whose expression is induced or suppressed by ABA treatment (Shen et al., 2010). Most of the ABA-responsive miRNAs (32) were responsive to at least one of the stress (drought, salt, or cold) treatments, suggesting that they are crucial for ABA-related stress pathways (Shen et al., 2010).

The current knowledge indicates that epigenetic modulation through DNA methylation, histone acetylation, and microRNA action, is critical to ABA-mediated stress signaling. A better understanding of epigenetics-mediated genome-environment interaction might provide new avenues for improving plant tolerance to abiotic stress.

## Crosstalk

### Crosslinks between ABA and MAPK Signaling Pathways

The mitogen-activated protein kinase (MAPK) cascade is an important signaling mechanism for regulating cellular responses to various stimuli including osmotic stress (Kelkar et al., 2000; Hamel et al., 2012). It is composed of an MAPK, MAPK kinase (MAPKK), and MAPKK kinase (MAPKKK). These three kinases can carry out phosphorylation relay from MAPKKK to MAPKK to MAPK. Twenty of MAPKs, 10 MAPKKs, and 80 MAPKKKs have been identified in *Arabidopsis* genome by structural and functional genome analysis (Colcombet and Hirt, 2008). A similar number of genes encoding MAPKs, MAPKKs, and MAPKKKs have been found in other plant’s genomes (Ichimura et al., 2002; Hamel et al., 2006; Colcombet and Hirt, 2008). For example, rice genome contains 17 MAPK genes, 8 MAPKK genes, and 74 MAPKKK genes (Reyna and Yang, 2006; Rao et al., 2010). Earlier study (Mizoguchi et al., 1996) reported that the MEKK1 (MAPKKK) transcript is up-regulated in response to various environmental stresses such as high salinity, low temperature, and mechanical stress. Teige et al. (2004), proposed the complete MAPK cascade functioning model MEKK1-MEKK2-MPK4/MPK6 for abiotic stress signaling. Few years later, some study showed that this model has a significant role in abiotic stress signaling (Ichimura et al., 2000; Teige et al., 2004; Nakagami et al., 2006; Ortiz-Masia et al., 2007; Xing et al., 2008). Many studies reported that MAPK cascades are involved in several ABA responses such as seed germination, guard cell signaling, and antioxidant defense (Xing et al., 2008; Jammes et al., 2009; Zhang et al., 2012).

It has been shown that ABA can affect the transcriptional regulation, protein accumulation, and stability of different component in MAPK cascade. For example, transcriptional expression of MAPKs (MPK3, MPK5, MPK7, MPK18, MPK20, MKK9), MAPKKKs (Raf6, Raf12, and Raf35), MAPKKK1(ANP1) and MAPKKK14-19 in *Arabidopsis* is induced by ABA treatment, suggesting that they play a significant role in ABA signaling. However, the functions of many MAPK pathway genes are still unknown (Menges et al., 2008; Wang et al., 2010; Danquah et al., 2014). Similarly in case of rice, ABA induces transcriptional activation of several MAPKs like OsMAP1(OsMAPK5), OsMAPK2, OsMSRMK2, OsMSRMK3, OsMAPK44, OsBIMK1, DMS1, OsEDR1, OsSIPK, OsWJUMK1 and OmMKK1 (Huang et al., 2002; Song and Goodman, 2002; Wen et al., 2002; Agrawal et al., 2003; Xiong and Yang, 2003; Jeong et al., 2006; You et al., 2007; Lee et al., 2008; De Vleesschauwer et al., 2010; Ning et al., 2010). Similar to *Arabidopsis* and rice, ABA also induces MAPKs in maize like ZmMPK7, ZmPK17, ZmSIMK1, and ZmMPK3 (Zong et al., 2009; Gu et al., 2010; Wang et al., 2010; Pan et al., 2012). Recently Zhang et al. (2012) reported that ZmMKK3 is involved in osmotic stress and ABA responses. In pea, p45MAPK is ABA-activated MAPK involved in guard cell signaling (Burnett et al., 2000; Schroeder et al., 2001). Brock et al. (2010) reported that a double knockout mutant (*pp2c5/ap2c1*) of the MAP-interacting phosphatases showed increased ABA-dependent activation of MPK3 and MPK6, which gives ABA sensitivity and suggests that MAPK cascade involving MPK3/MPK6 adversely regulates ABA signaling in plants.

Recently Danquah et al. (2015) demonstrated that a complete MAPK cascade (comprised of MAP3K17/18, MKK3, and the MAPKs MPK1/2/7/14) can be regulated by ABA core signaling module. They performed yeast two-hybrid analysis against all MAP2Ks and identified that only MAP2K is interacting with MAP3K17 and 18. All plants genome contain one gene coding for such an MKK3-like MAP2K, suggesting that this fusion occurred early and were conserved during evolution. Danquah et al. (2015) showed that a loss of function of MKK3 totally abolished ABA-dependent MPK17 activation, suggesting that this MAP2k is a significant factor of the ABA-triggered MAPK pathway. They also reported that ABA-triggered MAPK pathway depends on the transcription and protein synthesis of the MAP3Ks, MAP3K17, and MAP3K18. They also showed that ABA core machinery is required for the activation of the MAP3k17/18-MKK3-MPK1/2/7/14. The knowledge of crosslinks between MAPK cascade and ABA signaling should provide valuable information on the complex mechanism of plant stress adaptation, which helps to enhance crop stress tolerance that will be helpful for future sustainable agriculture.

### Crosstalk between ABA and Other Hormones

Abscisic acid and auxin control many aspects of plant growth and development together, mostly in opposing directions (Sun and Li, 2014). For examples, the expression of *AUXIN RESPONSE FACTOR 2* (*ARF2*) induced by ABA and *arf2* mutants exhibited enhanced ABA sensitivity in seed germination and primary root growth (Wang et al., 2011b). ABA treatment reduces the cell division and alters the auxin distribution more in *arf2* mutants as compared to wildtypes. Mutant *arf2* showed shorter roots under ABA application. They also suggested that there is a significant mechanism in ABA inhibiting the primary root growth through mediating cell division in root tips (Wang et al., 2011b). ABA inhibits seedling growth through enhancing auxin signaling (Belin et al., 2009). ABA and auxin also control seed dormancy through gene expression network involving ABSCISIC ACID INSENSITIVE 3 (ABI3) transcription factor (Liu et al., 2013). Auxin acts upstream of the major regulator of seed dormancy, ABI3, by recruiting AUXIN RESPONSE FACTOR 10 and AUXIN RESPONSE FACTOR 16 to control the expression of ABI3 during seed germination. They also showed that auxin is essential for seed dormancy and ABA inhibition of seed germination, signifying that the roles of auxin and ABA in seed dormancy are mutually dependent. In the ABA-mediated process, auxin functions in two ways: auxin activates the ABA response or stimulates the ABA biosynthesis response (Liu et al., 2013). In radical protrusion assayed, both exogenous and endogenous indole acetic acid (IAA) inhibited seed germination, indicating that ABA and auxin act synergistically to inhibit seed germination (Liu et al., 2013). They also observed that equal amount of IAA did not inhibit the seed germination in the absence of ABA, indicating that the auxin-mediated inhibition of seed germination is dependent on ABA. Further, osmotically induced ABA increases basipetal auxin transport through elevated AUX1 (an auxin influx transporter) and PIN2 (an auxin eﬄux transporter) levels (Xu et al., 2013). Therefore, ABA and auxin interact with crosstalk network via auxin response pathways in regulating plant growth.

Abscisic acid and cytokinin also appear to crosstalk each other (El-Showk et al., 2013). Cytokinin degradation is mediated by cytokinin oxidase enzymes which are encoded by CYTOKININ OXIDASE (CKX) multigene families with a varying number of members. For example, the *Arabidopsis* genome contains 7 CKX genes (*CKX1-CKX7*). Microarray and RT-PCR analysis showed that ABA downregulates CKX genes (*CKX1*, *CKX3*, *CKX4*, and *CKX6*) (Werner et al., 2006). During stress conditions, cytokinin receptor kinases (AHK2 and AHK3) negatively regulate ABA as well as osmotic stress responsive gene expression, whereas, cytokinin-deficient mutants have increased survival rates under stress (Tran et al., 2007). In addition, AHK2 and AHK3 are involved in mediating cold stress response by inhibiting ABA signaling (Jeon et al., 2010). Therefore, ABA and cytokinin have crosstalk to regulate stress responses.

Abscisic acid and ethylene interact to control the regulation of stomatal closure (Harrison, 2012). ABA and ethylene also control the increased ethylene concentration in leaves. The ethylene delays stomata closure by inhibiting the ABA signaling pathway (Tanaka et al., 2005). ABA inhibits ethylene biosynthesis by positively regulating ethylene response factor 11 (*ERF11*) via LONG HYPOCOTYL5 (HY5) transcription factor to repress expression of ethylene biosynthesis gene (*ACS5*), which encodes the rate-limiting enzyme in ethylene biosynthesis. The HY5 transcription factor serves as an important molecular link between ABA and ethylene biosynthesis (Li et al., 2011). They proposed a model suggesting that the HYF-AtERF11 regulon is a key factor modulating ABA-regulated ethylene biosynthesis (Li et al., 2011).

There is a complex interplay between ABA-signaling pathway and jasmonate (JA) regulating plant defense and gene expression (Anderson et al., 2004). For example, exogenous ABA treatment could suppress both basal and JA activated transcription from defense gene. In contrast, mutation of the ABA synthesis genes *aba1* and *aba2* resulted in upregulation of JA-responsive defense gene. These results indicate that the antagonistic interactions between multiple components of ABA and the JA-ethylene signaling pathways to modulate defense and stress-responsive gene expression in response to abiotic and biotic stresses (Anderson et al., 2004). Transcription factor MYC2 is a master regulator of most aspects of the JA-signaling pathway in *Arabidopsis* (Chen et al., 2011; Fernandez-Calvo et al., 2011). Transgenic plants overexpressing *MYC2* gene had higher sensitivity to ABA and several ABA-inducible genes upregulated in the transgenic plants whereas Ds insertion mutant *myc2* was less sensitive to ABA and showed reduced ABA-induced gene expression (Abe et al., 2003). Thus, MYC2 is proposed as an integrative hub for the regulation of both JA and ABA signaling in *Arabidopsis* (Sun and Li, 2014).

Thus, ABA is an important phytohormone which regulates plant growth and development in response to environmental stresses interacting through other plant hormones. The molecular mechanisms for the complex signaling networks between ABA and other hormones still need to study for further details in future.

## Conclusion and Future Perspectives

Due to growing population and global climate change food demand is increasing day by day. Thus, to fulfill the required demand, food production must be increased. Therefore, understanding abiotic stress tolerance in the plant has important, and broad spread implications that can not be understated. ABA is a vital hormone and acts as a central regulator of different plant stresses like drought, low temperature, and salinity. Due to the advancement of molecular genetic and genome-wide technologies, a deeper understanding of the underlying mechanisms of the involvement of ABA in stress tolerance is achieved, although more is yet to be revealed. The different physiological reactions that ABA regulates have been described at molecular level like stomatal closure, change in gene expression and accumulation of osmoprotectants. Understanding of ABA signal transduction pathways and epigenetic modifications can be further exploited to produce better transgenic plants with improved stress tolerance without a yield penalty that can withstand adverse climatic conditions. Genetic regulation through cis-engineering can be accomplished by designing promoter targeted at tissue-specific expression through which plant dynamics can be further explored. Many efforts are still required to uncover in details of each product of a gene induced by ABA and their interacting partners to understand the complexity of stress signal transduction pathways. The source of ABA in guard cells is still not fully understood. The mechanisms by which the abiotic stress upregulate ABA biosynthesis genes are still needed to know. Furthermore, the molecular mechanisms of crosstalk between ABA and other phytohormone signaling pathways remain to be elucidated. Although many questions are still open, the current advances in ABA signaling in *Arabidopsis* pave the way to address the molecular events underlying stress responses in other plant species, with the prospect to improve the abiotic stress performance of crop plants. Recently, Park et al. (2015) engineered ABA receptors by agrochemical engineering that provides the lots of opportunity in crop improvement program.

## Author Contributions

SS, JL, and KR drafted the manuscript. SS and JL designed the figures. SS, JL, and KR revised the manuscript.

## Conflict of Interest Statement

The authors declare that the research was conducted in the absence of any commercial or financial relationships that could be construed as a potential conflict of interest.
